# RNA-Seq reveals genotype-specific molecular responses to water deficit in eucalyptus

**DOI:** 10.1186/1471-2164-12-538

**Published:** 2011-11-02

**Authors:** Emilie Villar, Christophe Klopp, Céline Noirot, Evandro Novaes, Matias Kirst, Christophe Plomion, Jean-Marc Gion

**Affiliations:** 1 CIRAD, UMR AGAP, Campus de Baillarguet TA 10C, F-34398 Montpellier Cedex 5, France; 2 INRA, UMR1202 BIOGECO, F-33610 Cestas, France; 3 CRDPI, BP1291, Pointe Noire, République du Congo; 4 Plateforme bioinformatique Genotoul, UR875 Biométrie et Intelligence Artificielle, INRA, 31326 Castanet-Tolosan, France; 5 School of Forest Resources and Conservation, University of Florida, PO Box 110410, Gainesville, USA; 6 Universidade Federal de Goiás, Caixa Postal 131, CEP 74690-900, Goiânia, Brazil; 7 Université de Bordeaux, UMR1202 BIOGECO, F-33610 Cestas, France

## Abstract

**Background:**

In a context of climate change, phenotypic plasticity provides long-lived species, such as trees, with the means to adapt to environmental variations occurring within a single generation. In eucalyptus plantations, water availability is a key factor limiting productivity. However, the molecular mechanisms underlying the adaptation of eucalyptus to water shortage remain unclear. In this study, we compared the molecular responses of two commercial eucalyptus hybrids during the dry season. Both hybrids differ in productivity when grown under water deficit.

**Results:**

Pyrosequencing of RNA extracted from shoot apices provided extensive transcriptome coverage - a catalog of 129,993 unigenes (49,748 contigs and 80,245 singletons) was generated from 398 million base pairs, or 1.14 million reads. The pyrosequencing data enriched considerably existing *Eucalyptus *EST collections, adding 36,985 unigenes not previously represented. Digital analysis of read abundance in 14,460 contigs identified 1,280 that were differentially expressed between the two genotypes, 155 contigs showing differential expression between treatments (irrigated vs. non irrigated conditions during the dry season), and 274 contigs with significant genotype-by-treatment interaction. The more productive genotype displayed a larger set of genes responding to water stress. Moreover, stress signal transduction seemed to involve different pathways in the two genotypes, suggesting that water shortage induces distinct cellular stress cascades. Similarly, the response of functional proteins also varied widely between genotypes: the most productive genotype decreased expression of genes related to photosystem, transport and secondary metabolism, whereas genes related to primary metabolism and cell organisation were over-expressed.

**Conclusions:**

For the most productive genotype, the ability to express a broader set of genes in response to water availability appears to be a key characteristic in the maintenance of biomass growth during the dry season. Its strategy may involve a decrease of photosynthetic activity during the dry season associated with resources reallocation through major changes in the expression of primary metabolism associated genes. Further efforts will be needed to assess the adaptive nature of the genes highlighted in this study.

## Background

Planted forests constitute only 7% of the global forested area, but contribute to a significant proportion of overall forest goods and services (e.g. up to 35% of industrial roundwood supply). In the context of climate change, the adaptation of planted forests is essential for a sustainable forestry sector. The adaptation of industrial plantations to present and future environmental conditions (including extreme weather events) depends on several factors, including the genetic diversity of the material used for reforestation and the phenotypic plasticity of individual genotypes. Genetic diversity ensures that forest trees can survive, adapt and evolve under changing environmental conditions [1,2], whereas phenotypic plasticity constitutes a shorter term response to environmental changes at the individual level of particular importance in long-lived organisms, such as trees [2,3].

*Eucalyptus *is one of the key genera among planted trees. The genus is includes the most important hardwood fibre crops species planted worldwide (19 million hectares according to [4]). Several *Eucalyptus *species grow rapidly and are highly adaptable. These properties led to their introduction worldwide, at latitudes extending from southern Europe to South Africa. In its natural range (Australia and some nearby islands), *Eucalyptus *are also found in a diverse spectrum of ecological niches. The genetic diversity of *Eucalyptus *has been studied extensively and remarkable levels of variation have been detected using neutral markers [5-11] and in genes possibly involved in adaptive traits [12-14]. Phenotypic plasticity is also likely to ensure better adaptation of individual genotypes to changing environmental conditions [15] and is of particular importance in clonal forestry.

Ecophysiological studies have shown that water is the principal factor limiting stem growth in *Eucalyptus *(e.g. [16,17]). Moreover, some studies have reported that eucalyptus genotypes differ in terms of their capacity for phenotypic modification in response to water deficit [18-20]. Several physiological mechanisms for coping with drought have been described in these species: i) the regulation of transpiration to decrease water loss [21], ii) resource reallocation from the shoot to the root, to increase water uptake [17], and iii) adjustment of osmotic potential [22] or protection against reactive oxygen species, to prevent damage due to stress [20]. Drought tolerance mechanisms have been described in detail at the molecular level for both annual and perennial model plants, such as *Arabidopsis *[23-25] and *Populus *[26-28], but little is known about the molecular basis of drought tolerance in *Eucalyptus*, particularly in field conditions.

Next-generation sequencing (NGS) provides new opportunities for studies of the molecular plasticity in response to water deficit. The high throughput of NGS is particularly useful in non-model organisms for which few genomic resources are available [29]. Moreover, NGS is suitable for transcript profiling, combining the high throughput of serial analysis of gene expression (SAGE) with the functional annotation capacity of EST sequencing [30]. These techniques have been widely used for transcriptome profiling, particularly for studies of biotic [31] and abiotic [24] stress responses, and the characterisation of developmental processes [32]. Considerable sequencing depth can be obtained, making it possible to identify transcriptome expression variation [29].

In plants, the shoot apical meristem (SAM) is a key organ in stem development. The SAM initiates phytomers and regulates shoot growth by integrating several signals, such as hormones (ABA, auxins, cytokinins) and transcription (e.g. homeobox) [33]. When plants are subjected to environmental stimuli, the leaf developmental network is adjusted by changes in shoot apex activation [34]. In *Eucalyptus*, EST resources have been developed for various tissues, such as roots, leaves and wood-forming tissues [35-38], but a limited number of genomic resources are available for shoot apices, despite the important role of this organ in plant organogenesis.

In this study, we compared transcript profiles in the shoot apices of two eucalyptus genotypes used in industrial plantations, under two watering regimes -- irrigated (IR) *versus *non-irrigated (NI). The two genotypes differ in their growth rates and ecophysiological characteristics at maturity, with one genotype being more productive and water use-efficient than the other. We used pyrosequencing (Roche 454) to sequence non-normalized cDNA libraries constructed from shoot tip mRNA. After verifying technical reproducibility, we addressed the following questions: i) Are there molecular differences between genotypes, reflected in the contrasting phenotypes, and do these differences affect specific pathways or have a random effect on the transcriptome? ii) Can we detect molecular plasticity in the response to water shortage during the dry season, and which pathways are affected? iii) Does this plasticity differ between genotypes (i.e. is there any genotype-by-environment interaction?), and which genes or pathways reflect these differences?

## Methods

### Plant material

We compared the response of two eucalyptus genotypes, 1-41 (NCBI Taxonomy ID: 764271) and 18-50 (NCBI Taxonomy ID: 765255), to water shortage during the dry season of 2008. These two genotypes are used in industrial plantations in the Republic of Congo. Hybrid 1-41 (named G1 in the following sections) was obtained by open pollination of *E. alba *(the male parent is unknown) and the hybrid 18-50 (named G2) was derived from a controlled pollination of *E. urophylla *(genotype 14-36) by *E. grandis *(genotype 9-10). These two hybrids differ in their growth rates and water use efficiency (WUE, estimated by isotopic carbon composition) at maturity, G2 being superior than G1.

### Field experiment

Trees were vegetatively propagated by rooted cuttings and established in a field experiment in Yanika, Republic of Congo (4°20'S, 11°38'E, 50 m above sea level), in June 2007. Trees were planted in plots of 64 cuttings per genotype and per treatment, including a buffer zone of 40 plants. Two watering regimes were used during the dry season: no irrigation (NI) and irrigation (IR). Trees were watered with sprinklers, to replenish evapotranspiration losses, estimated at 3 mm per day. In order to evaluate the effect of water deficit on above-ground biomass growth and molecular plasticity, plant material was sampled in September 2008, 16 months after the trees were planted. The dry season began approximately on May 15^th ^2008 -therefore, trees under NI treatment were subjected to four months without rainfall by the time samples were collected.

### Soil water content

Volumetric water content (VWC) was measured by time domain reflectometry (TDR; Trase system, Soil moisture, Santa Barbara, CA). Four series of TDR probes per genotype and per treatment were installed horizontally, at six depths (0.15, 0.5, 1, 2, 3 and 4 m). Mean values were calculated from the four replicated measurements at each depth.

### Biomass production

We harvested 11 trees per treatment and dissected them into the following compartments: stem, dead branches, living branches and leaves. Each compartment was weighed in the field. Representative subsamples of each compartment were then harvested, and weighed before and after drying at 65 °C to constant weight. Water content was calculated for each of these subsamples and used to estimate total dry biomass for each compartment. Total above-ground biomass (the sum of all the compartment) was analysed by two-way ANOVA, with R (R Development Core Team), according to the following model:

$$X_{ijk}=\mu +a\cdot G_{i}+b\cdot T_{j}+c\cdot {\left(G\times T\right)}_{ij}+\varepsilon_{ijk}$$

where X_ijk _is the above-ground biomass in genotype *i *(G1 or G2), treatment *j *(NI or IR) and replicate *k. a, b *and *c *are the regression coefficients of *G*, the genotypic effect, *T*, the treatment effect and *G *× *T *the interaction between genotype and treatment, and ε_ijk _is the residual.

### cDNA synthesis

**Figure 1:**
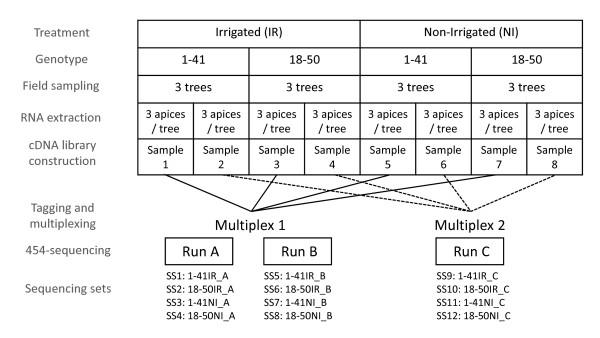
**Procedure used, from tissue sampling to sequencing**. Two genotypes (G1 and G2) were subjected to two watering regimes (IR and NI). Shoot apices from three trees per genotype and per treatment were collected in the field. Total RNA was extracted from three apices per tree. Two replicate RNA extractions were carried out for the construction of two independent replicate cDNA libraries per genotype and per treatment (resulting in 8 templates for cDNA library construction). For sequencing, each cDNA library was tagged, and two independent multiplexes were created by pooling one sample for each genotype and treatment combination. Multiplex #1 was sequenced with two 454-Roche FLX Titanium half-runs, resulting in eight sequencing sets, whereas multiplex #2 was sequenced with one half-run and resulted in four sequencing sets.

The experimental design, from tissue sampling to library construction and sequencing, is described in figure 1. Shoot apices were collected from three trees from each genotype and treatment, and immediately frozen in liquid nitrogen. Two RNA extractions of three apices from each tree were performed, as previously described by Reid *et al*. [39]. RNA samples were treated with Turbo RNase-Free DNase (Ambion, Austin, TX, USA) and purified with the RNeasy Plant Mini Kit (Qiagen, Valencia, CA, USA). RNA concentration and quality were analysed with an Agilent Technologies 2100 Bioanalyser (Agilent Technologies, Mississauga, ON, USA) and a ND-1000 Spectrophotometer (NanoDrop, Wilmington, DE, USA). The three RNA preparations per replicate and per condition (corresponding to three trees), were pooled in equal proportions, providing templates for cDNA libraries S1-S8 (figure 1). Full-length cDNA was obtained from 1 μg of RNA, with the Smart cDNA Library Construction Kit (Clontech, Mountain View, CA, USA), according to the manufacturer's instructions. We amplified the cDNA with PCR Advantage II Polymerase (Clontech, Mountain View, CA, USA), over 16 cycles (7 s at 95 C, 20 s at 66 °C, and 4 min at 72 °C). This cDNA amplification procedure was repeated eight times in separate tubes for each sample, with pooling to give a total of 6 μg of cDNA fragments longer than 1,000 bp quantified with an Agilent Technologies 2100 Bioanalyzer. Eight cDNA libraries (S1-S8) were constructed, giving two biological replicates for the two genotypes (G1, G2) subjected to the two watering regimes (IR, NI).

### Library construction, 454-sequencing, sequence quality control and assembly

**Figure 2:**
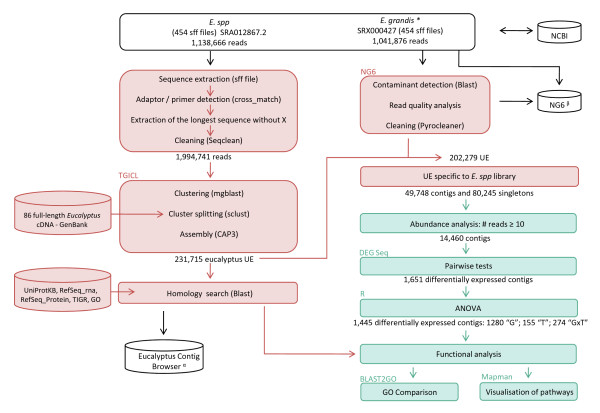
**Pipeline used for sequence analysis**. White boxes correspond to sequence generation, red boxes correspond to library quality control, assembly and annotation and green boxes correspond to abundance and functional analysis. The software suite or program used is indicated in the upper part of the box and the results of each step of the analysis are shown under the box. UE: unigene element; "G": genetic effect, "T": treatment effect and "GxT": genotype by treatment interaction effect. *E. grandis. sff files were obtained from Novaes *et al*. (2008). α: available at http://genotoul-contigbrowser.toulouse.inra.fr:9092/Eucalyptus/index.html β: available at http://vm-bioinfo.toulouse.inra.fr/ng6/, user = euca; password = euca33bio!

We nebulised 5 μg of each cDNA sample to a mean fragment size of 650 bp and ligated it to an adaptor, according to standard procedures [40]. Each cDNA library was tagged with Multiplex Identifiers (MID) barcode adaptors, and two independent multiplexes were created by pooling one sample from each genotype and treatment. Multiplex #1 comprised samples S1, S3, S5 and S7, whereas muliplex #2 comprised samples S2, S4, S6 and S8. One half-run (run A) of sequencing was initially carried out for multiplex #1 on a GS-FLX Titanium platform (454 Life Science, Brandford, CT, USA) at Cogenics (Meylan, France). Two half-runs of sequencing for multiplex #1 (run B) and multiplex #2 (run C) were then performed by Agencourt (Beverly, MA, USA) on a GS-FLX Titanium sequencer. Base calling with GS-FLX System software generated 353,344 high-quality reads for the first half-run and in 785,322 reads for the second complete run. Sequences were deposited at the NCBI short-read archive (SRA) under accession number SRA012867.2 (Figure 2). The 454-sequencing reads (1,138,666 from this study and 1,041,876 from Novaes *et al*. [13]) were screened by cross_match (http://bozeman.mbt.washington.edu/phrap.docs/phrap.html) for primers and adaptors and then masked. For each 454-sequencing read, the longest non-masked region was extracted and further trimmed with SeqClean (http://compbio.dfci.harvard.edu/tgi/). The shorter regions were discarded to eliminate potential chimeras. Sequences were assembled as previously described [41], with TGICL [42], using the 12 sets of sequencing data from this study and the four sets of sequencing data obtained for *E. grandis *[accession number SRA001122] by Novaes *et al*. [13]. In parallel, all reads were stored in the NG6 system (http://vm-bioinfo.toulouse.inra.fr/ng6/, project: BIOGECO eucalyptus) and three kinds of analysis were performed for each of the 16 sequencing sets, as previously described [41]: i) BLAST search for *E. coli*, phage and yeast contaminants, ii) read quality analysis and iii) removal of sequences that were too long or too short, sequences with an excess of errors (more than 4% of N), low-complexity sequences and duplicated reads, using Pyrocleaner program. Only unigene elements (UE) resulting from sequences generated in this experiment (the *E.spp *sequencing set) were used for digital gene expression analysis.

### Digital gene expression analysis

Contigs with less than 10 reads for the 12 sequencing sets generated in this study were eliminated from further statistical analyses. For the 14,460 remaining contigs, the numbers of reads per sequencing set and per contig were used to assess gene abundance. Two types of statistical analysis were performed. First, pairwise comparisons were carried out between genotypes (G2 vs. G1 sequencing sets, irrespective of treatment) and between treatments (IR vs. NI sequencing sets, irrespective of genotype). Four additional comparisons were carried out for each genotype and each treatment, as follows: IR vs. NI for genotypes G1 and G2, G1 vs. G2 for treatments IR and NI. Statistical tests, based on the use of the MARS method in the DEGseq package [43] were performed to assess differential expression [43]. Second, two-way analysis of variance (ANOVA) was performed on contigs, making use of the three replicates (run A, B and C) per treatment to estimate random variation and test the genotype (G), treatment (T) and genotype-treatment interaction (GxT) effects. Transcript abundance was normalized by dividing the number of reads by the sequence length of the contigs and the total number of sequences in each sequencing set. Contigs with a q-value <0.05 in the DEGseq test (i.e. after false discovery rate corrections) [44] and with p-values <0.05 after ANOVA were considered to be differentially expressed and were extracted for further analysis. The 14,460 genes analysed were classified into four classes: not significantly differentially expressed (NS), and showing genotype ("G" contigs), treatment ("T" contigs) or genotype × treatment ("GxT" contigs) effects. For the comparison of expression levels, we used log_2_-transformed fold-changes between contig abundances in the various contrasts obtained in the DEGseq analysis.

### Functional annotation of differentially expressed genes

Contigs were assigned a putative function by BLASTX [45], using various public databases: UniProtKB/Swiss-Prot (release 57.1), RefSeq Protein (release 34), Pfam (release 23.0), with an e-value cut-off value of 10e^-5^. Sequences were also compared to TIGR's assemblies of Arabidopsis_thaliana (release 14), Helianthus_annuus (release 6), Populus (release 4), Picea (release 3) and Vitis vinifera (release 6), with an e-value cut-off of 10e^-2^. Gene Ontology terms were assigned via the UniprotKB accession and clustered with Blast2GO [46]. The differential distributions of each class of effect (T, G, GxT and NS) between Biological Processes, Molecular Functions and Cellular Components were assessed using Fisher's exact tests, with a significance threshold of 0.05. Pathway analysis was carried out with Mapman [47]. Differentially expressed contigs were assigned to functional categories (or bins) by Mercator (http://mapman.gabipd.org/web/guest/mercator). A dedicated pathway map was created to represent most of these contigs. The Wilcoxon rank sum test was used to identify differentially regulated bins.

## Results

### Monitoring of soil water content

A factorial design including two genotypes (G1 and G2) and two water regimes (irrigated IR vs. non-irrigated NI) was established in a field trial in one of the main areas of eucalyptus plantation in the Republic of Congo. The experiment was evaluated over a period of two years. Soil water content (SWC) was monitored throughout the experiment at six depths (0.15-4 m), to assess water availability in different experimental conditions. In this study, we focus on the effect of water availability in the second dry season (after four months without rainfall) on biomass production and the transcriptome.

**Figure 3:**
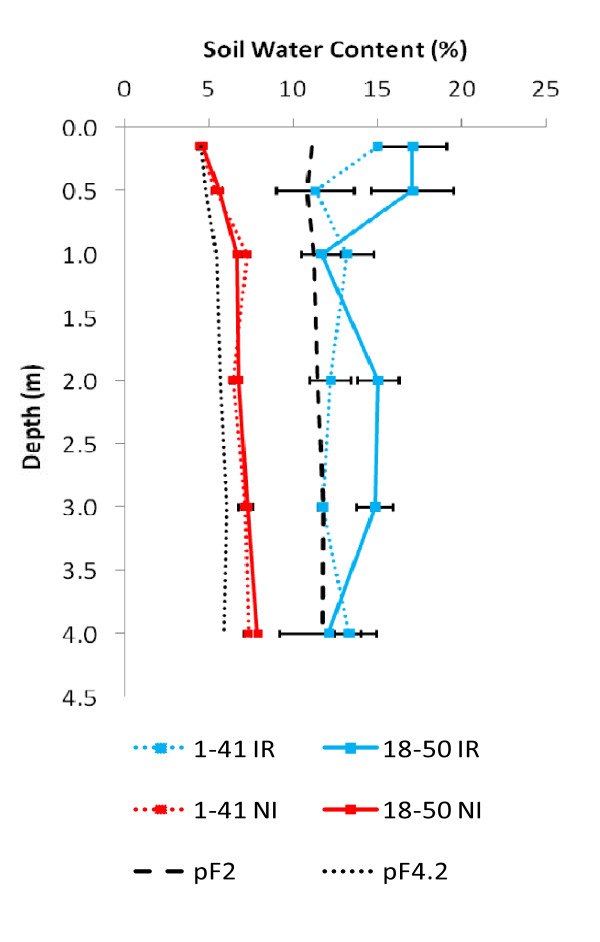
**Profile of soil water content (SWC)**. Means and standard errors of SWC at six depths, for both genotypes (G1 and G2), subjected to two water regimes (irrigated: IR and not irrigated: NI). SWC at wilting point (pF4.2) and at field capacity (pF2) were calculated from data obtained at a site close to the experimental field (Laclau, personal communication).

In the NI treatment, SWC varied from 4.5% to 8%, and no significant difference was found between the two genotypes (Figure 3). SWC values were close to wilting point (pF 4.2), i.e., when plants ceased to be able to absorb soil water. In the IR treatment, SWC ranged from 11% to 17%, and exceeded field capacity (pF 2.0), indicating that water was not a limiting factor for tree growth. SWC was also higher in the area surrounding genotype G2 than in genotype G1, except at two depths (1 m and 4 m). This result suggests that the two genotypes absorb water preferentially from different depths, possibly because their root system develops differently.

### Effect of water deficit on biomass production

**Figure 4:**
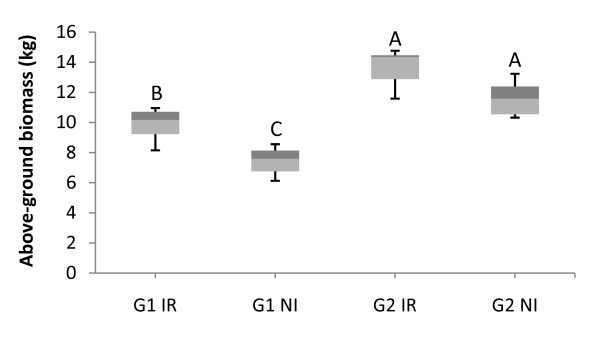
**Above-ground biomass**. Box plot of above-ground biomass calculated for 11 trees per treatment, for both genotypes (G1 and G2), subjected to two water regimes (irrigated: IR and not irrigated: NI). Centre line and outside edge of each box indicate the median and range of inner quartile around the median, respectively; vertical lines on the two sides of the box represent the first and the ninth decile, respectively. Letters indicate the groups obtained in Bonferroni tests for multiple pairwise comparisons.

Genotype (p < 0.0001) and treatment (p < 0.001) had significant effects on above-ground biomass production (stem, dead branches, living branches and leaves) (Figure 4). Mean biomass was much higher for genotype G2 (12.8 kg) than for genotype G1 (8.6 kg) confirming earlier findings [48]. Not surprisingly, mean biomass was higher for the IR treatment (11.8 kg) than for the NI treatment (9.7 kg). A two-way ANOVA showed that the GxT interaction effect was not significant (Additional file 1), but relative biomass loss was nonetheless lower for genotype G2 (12%) than for genotype G1 (24%). These results suggest that the growth of genotype G2 is less affected by water shortage compared to G1.

### Sequencing of the *Eucalyptus *shoot apex transcriptome

We chose to sequence the shoot apex transcriptome to study the molecular response of eucalyptus to water deficit, due to its role in shoot organogenesis [49]. Shoot apices were pooled from three trees in four sets of conditions (2 genotypes × 2 treatments). Three half-runs (A, B, C) of 454-Roche FLX Titanium sequencing provided 353,344 (run A), 405,223 (run B) and 380,099 (run C) reads, for a total of 1,138,666 sequences (398 Mb). The mean read length was 334 bp for run A, 369 bp for run B and 344 bp for run C (Table 1). Reads were slightly shorter for run A, with a higher abundance of reads comprised between 400-420 bp in length, whereas runs B and C were characterised by reads of 460-480 bp in length (Additional file 2). To increase contig length of the assembly, we combined all the reads (1,138,666) generated in this study (*E. spp *sequencing set) with other GS-20 and GS-FLX 454 reads (1,041,876 reads) from various organs of *E. grandis *(*E. grandis *sequencing set; [13]). Figure 2 summarises the various stages in sequence analysis, corresponding to data quality control, read assembly, annotation and abundance analysis. After removal of vector and adaptor sequences, 1,994,741 reads were available for assembly. Assembly with TGICL generated 231,715 unigene elements (UE) comprising 80,854 contigs and 150,861 singletons. Removal of low-quality sequences and duplicated reads with the NG6 platform resulted in a total of 202,279 UE (69,584 contigs and 132,690 singletons), from which 129,993 UE (49,748 contigs and 80,245 singletons) were identified in the *E. spp *sequencing set (this study) and used for digital gene expression analysis (Table 2).

**Table 1:** Summary statistics for the three 454-sequencing half-runs

Sample	Genotype	Treatment	Sequencing set	Runs	# of Reads	# of Reads	# of bp	Average length of reads (bp)	Average length of reads (bp)
1	G1	IR				58,921	19,313,318	328	
3	G2	IR	SS2: G2IR_A			92,165	30,365,092	329	
5	G1	NI	SS3: G1-NI_A	run A	353,344	95,500	31,350,808	328	334
7	G2	NI	SS4: G2NI_A			106,758	37,126,644	348	

1	G1	IR	SS5: G1IR_B			139,137	51,085,203	367	
3	G2	IR	SS6: G2IR_B	run B	405,223	112,051	41,110,040	367	
5	G1	NI	SS7: G1-NI_B			59,907	21,833,839	364	369
7	G2	NI	SS8: G2NI_B			94,128	35,374,789	376	

2	G1	IR	SS9: G1IR_C			93,651	32,338,261	345	
4	G2	IR	SS10: G2IR_C			90,511	31,595,445	349	
6	G1	NI	SS11: G1NI_C	run C	380,099	83,530	27,929,481	334	344
8	G2	NI	SS12: G2NI_C			112,407	38,802,862	345	

TOTAL				1.5 run GS-FLX Titanium	1,138,666	1,138,666	398,225,782	350	350

**Table 2:** Assembly statistics from TGICL (I), and figures obtained after Pyrocleaner analysis (II), for the set of sequences reported here (III)

	TGICL (I)	Pyrocleaner (II)	*E. spp *(III)
# Contigs	80,854	69,584	49,748
# reads in contigs	1,843,806	1,386,859	851,751
Average length of all contigs (bp)	552	608	734
# Large Contigs >500 bp	34,076	33,962	32,694
Average length of large contigs	900	901	912
# Singletons	150,861	132,695	80,245
# UE	231,715	202,279	129,993

### Contribution of the three half-run replicates

In total, 90,579 UE (70% of *E. spp *UE) did not have sequence similarity to the *E. grandis *sequence reads from Novaes *et al*. [13]. Most of these sequences were singletons (71,761), although some were contigs (18,818), corresponding to 27% of the *E. spp *contigs. A BLAST homology search (cut-off: 10^-10^) of published eucalyptus databases (ESTs from GenBank available on April 2010; 454-ESTs generated by Novaes *et al*., [13]; 454-ESTs from JGI from *E. globulus *xylem and leaf tissues; Illumina contigs generated by Mizrachi *et al*., [38]) showed that 21,401 UE (comprising 3,066 contigs and 18,335 singletons) did not match any known sequence. Thus, the resource described here greatly extends the list of genes known to be expressed in *Eucalyptus*, which will be critical for the annotation of the genome sequence. Due to the smaller number of reads in run A, the total number of UE including reads generated from run A was also smaller (58,763) than that generated from the other two runs (67,467 in run B and 67,756 in run C). Each supplementary half-run produced between 16 and 21% new contigs for a second half-run, and between 5 and 7% for a third half-run (Additional file 3). Vega-Arreguin *et al*. [50] reported similar trends in maize, with a plateau of gene representation reached after the third successive GS-20 454-sequencing run. The number of reads generated was therefore considered sufficient to sample most expressed genes.

**Figure 5:**
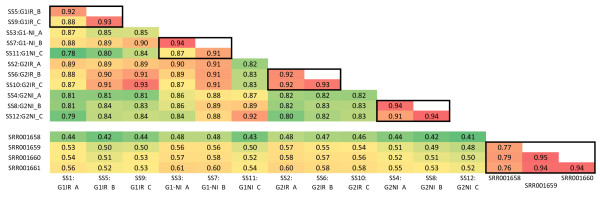
**Pearson correlation coefficient for read frequencies (within contigs) between sequencing sets**. SS1-SS12 are sequencing sets from *E.spp*; SRR001658-SRR001661 are sequencing sets from *E. grandis *(Novaes *et al.*, 2008). The colour scale indicates the strength of the correlation, from red (strongest correlations) to green (weakest correlations). All the correlation coefficients were significant, with a p-value <0.001.

The Pearson's correlation coefficients (figure 5) obtained for read frequencies in the 12 sequencing sets from *E. spp *(87% on average) and the four sequencing sets from *E. grandis *(86% on average) were similar. However, the mean correlation was much weaker (52%) between the *E. spp *and *E. grandis *sequencing sets, suggesting that different fractions of the transcriptome had been sampled from these two studies and/or that gene expression differed between both sequencing sets. As expected, correlations were stronger between replicates (92%; illustrated by squares in figure 5) than between different samples (86%**) **in *E. spp *sequencing sets, suggesting a high level of technical repeatability.

Within conditions, correlations between the G1 and G2 sequencing sets were robust and similar between treatments: 90% for the IR treatment and 88% for the NI treatment, suggesting that these two genotypes displayed similar patterns of gene expression when placed in the same environmental conditions. Correlations between the IR and NI sequencing sets were slightly weaker and a stronger contrast was observed within genotypes: on average, 86% for genotype G1 and 82% for genotype G2. The weaker correlations obtained for G2 suggest that this genotype had a more pronounced response to water deficit than genotype G1.

### Homology search

BLAST search (e-value cut-off of 10e^-5^) results are summarised in Table 3. A functional annotation was obtained more frequently for contigs - 70% of the contigs harboured similarity to sequences in protein databases, and 75% to sequences in nucleic acid databases. In contrast, for singletons only 39% and 9% of the contigs had similarity to protein and nucleic acid sequences, respectively. These differences were expected, given the longer mean length of the contigs (734 bp) compared to singletons (319 bp), their lower abundance and the fact that singletons are more likely to be sequencing artefacts. A larger number of nucleic acid sequences had similarity to *Arabidopsis *(55% of annotated sequences), followed by *Vitis *(25%), and *Populus *(6%). The greater similarity to *Arabidopsis *genes may be due to the closer phylogenetic relationship of *Eucalyptus *to *Arabidopsis *(both belong to the eurosid II phylogenetic clade) than to *Populus *(eurosid I, [51]). The *Arabidopsis *genome has also been annotated in greater detailed than the *Populus *genome. Interestingly, the similar characteristics of eucalyptus and poplar in terms of growth habits do not translated into higher similarity of the sequences transcribed.

**Table 3:** Annotation results for protein hits, nucleic acid hits, and Gene Ontologies (GO): Biological Process (BP), Cellular Component (CC) and Molecular Function (MF)

	*E.spp *Protein hits	SSequencing set	Nucleic acid hits	GO-BP	GO-CC	GO-MF
# UE	129,993	66,135 (51%)	44,652 (34%)	32,835 (25%)	30,836 (24%)	33,222 (26%)
# Contigs	49,748	34,951 (70%)	37,383 (75%)	18,091 (36%)	17,242 (35%)	18,355 (37%)
# Singletons	31,184 (39%)	80,245	7,269 (9%)	14,744 (18%)	13,594 (17%)	1,4867 (19%)

% of sequences related to E. spp. libraries is shown in brackets.

According to Gene Ontology (GO) classification, 38,190 UE (25% of the *E.spp *sequencing set UE) were associated with at least one biological process (BP), molecular function (MF) or cellular component (CC). The proportions of UE annotated in each category were generally similar to those obtained in *Arabidopsis *(Additional file 4), suggesting that the *E.spp *sequencing sets are appropriate for the analysis of gene expression on a broad range of functional categories.

### Transcript abundance analysis

**Figure 6:**
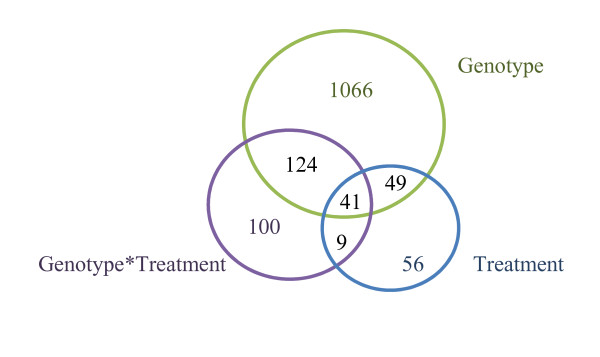
**Differentially expressed contigs**. Venn diagram indicating the number of differentially expressed contigs showing a G, T and/or GxT effect, for the 14,460 contigs analysed.

After removing contigs represented by fewer than 10 reads in all the *E.spp *sequencing sets, 14,460 contigs remained for abundance analysis. Two statistical tests were performed in series to detect differences in the expression levels of these 14,460 contigs, among the four experimental conditions (G1IR, G1NI, G2IR and G2NI). First, a DEG-seq test [43] identified 1,651 differentially expressed contigs (FDR ≤ 5%). A two-way ANOVA was then performed to assess the effects of the two main factors (G and T) and their interaction (GxT) on the number of reads per contig. This analysis identified 1,445 contigs with at least one significant effect (p-value ≤ 5%; figure 6, additional file 5). With an error rate of 5%, only 83 false positives are expected among the 1,445 contigs.

**Figure 7:**
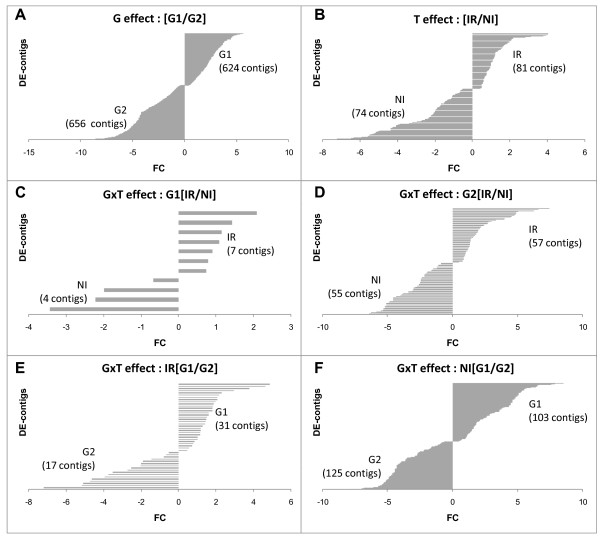
**Fold-change (FC) distribution**. Log_2_-transformed FC were calculated with DEGseq and plotted for each class of effect. (A) Genotype effect: contigs overexpressed in genotype G1 are presented with positive values (+), whereas contigs underexpressed in genotype G1 have negative values (-). Similarly, (B) treatment effects: overexpressed in IR (+) and under expressed in IR (-); (C) genotype × treatment effect for genotype G1: IR (+) and NI (-); (D) genotype × treatment effect for genotype G2: overexpressed in IR (+) and underexpressed in IR (-); (E) genotype × treatment effect for treatment IR: overexpressed for G1 (+) and underexpressed for G1 (-); (F) genotype × treatment effect for treatment NI: overexpressed for G1 (+) and underexpressed for G1 (-). The number of contigs overexpressed is shown in brackets.

Most of the differentially expressed contigs (1,280) showed a genotype effect ("G contigs"), with 624 "G contigs" overexpressed in genotype G1 (positive log_2_-transformed fold-change between contig abundance in G1 vs. G2) vs. 656 "G contigs" underexpressed in G1 ( negative log_2_-transformed fold-change; figure 7A). Of the 656 contigs overexpressed in genotype G2, 289 (44%) were expressed only in that genotype (with no corresponding reads in the G1 sequencing sets) whereas only 55 contigs (9%) showed the reverse trend, suggesting that G2 may express of a larger set of genes or different splicing variants.

A total of 155 contigs showing a treatment effect ("T contigs"; figure 7B) were identified with similar numbers overexpressed in the two treatments (81 in IR and 74 in NI). Thirteen "T contigs" were expressed only in NI conditions, whereas all the "T contigs" overexpressed in IR conditions were also found in NI sequencing sets, suggesting that few "specific genes" are upregulated in response to water deficit but that the set of genes expressed in favourable conditions is also expressed at a lower level in stressed plants.

Finally, 274 contigs corresponded to "GxT" contigs. The larger number of "GxT" contigs than of "T contigs" suggests that some of the observed molecular plasticity is under genetic control. Only 11 "GxT contigs" displayed significant differential expression between the IR and NI conditions in genotype G1 (figure 7C), whereas 112 "GxT contigs" displayed such behaviour in genotype G2 (figure 7D), suggesting a more pronounced response in G2. Similarly, 48 "GxT contigs" were differentially expressed between the two genotypes in IR conditions (figure 7E), whereas 228 "GxT contigs" were differentially expressed between the two genotypes for the NI treatment (figure 7F). These results suggest that, despite the rather similar expression patterns for the two genotypes in IR conditions, water deficit induced a molecular response specific to each genotype, reflecting different strategies to respond to water shortage during the dry season.

### Blast2GO functional analysis

**Figure 8:**
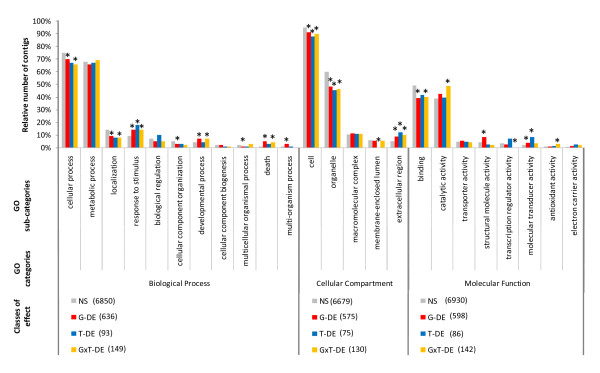
**Distribution of Gene Ontology categories between effect classes**. Percentage of contigs annotated for Biological Process, Cellular Component and Molecular Function GO category (level 2). Non-significant contigs (NS) and contigs displaying genotype (G), treatment (T), or genotype × treatment (G*T) effects are shown. The numbers in brackets are the numbers of annotated contigs per class of contigs and GO category. *Indicates distributions significantly different between NS and other classes of effect, in Fisher's exact tests with a threshold of 0.05.

Gene Ontology (GO) analysis was performed on 9,058 contigs (of 14,460 contigs containing more than 10 reads). These contigs were assigned to biological processes (BP), with 11 main subcategories (7,593 contigs), cellular compartments (CC), with five main subcategories (7,347 contigs), and molecular functions (MF), with eight main subcategories (7,638 contigs). Consistent with the number of annotations per category (BP, CC, MF), two subcategories were found to be strongly represented: > 65% of the contigs were assigned to cellular and metabolic processes for BP, more than 40% were assigned to cells and 85% to organelles for CC, and > 40% were assigned to binding and catalytic activity for MF. Figure 8 shows the distribution of contigs between these subcategories as a function of the four classes of effects ("NS", "G", "T", and "GxT" contigs). The homogeneity of the relative abundance of contigs between the "significant" classes ("G", "T", or "GxT") and the "not significant" class ("NS") in each GO category was assessed with Fisher's exact tests. In BP, "G" contigs were overrepresented in four subcategories (response to stimulus, developmental process, death and multiorganism process). "T" contigs were overrepresented in only one subcategory (response to stimulus). Finally, "GxT" contigs were overrepresented in three subcategories (response to stimulus, death and developmental process). These differences in relative abundance suggest that genes related to defence reactions are the main contributors to differences between significant and "NS" contigs. For CC, only one subcategory (extracellular region) presented a higher relative abundance for all three significant effects. For MF, "G" contigs were overrepresented in two subcategories (structural molecule activity and molecular transducer activity), whereas "T" contigs were overrepresented in only one subcategory (molecular transducer activity); for "GxT" contigs, two other subcategories (catalytic activity and antioxidant activity) presented higher relative abundances than "NS".

### Analysis of metabolic pathways with MapMan

Of the 1,445 contigs displaying significant differential expression, the 1,280 "G contigs" did not enable characterisation of specific molecular processes (i.e. did not show any clear co-regulation with genes of the same biosynthesis pathway). For 95% of these contigs, many different genes from different molecular processes were activated, depending on the experimental condition (Additional file 6). In some instances (5% of "G" contigs), some bins presented specific overexpression in one genotype (Additional file 7). For example, contigs related to ethylene biosynthesis and cell organisation were overexpressed mainly in genotype G2, whereas contigs related to photosynthesis, nitrilases, calcium signalling and pathogenesis-related protein bins were overexpressed in genotype G1. Serine proteases (9 contigs) were expressed more strongly in genotype G1 (p-value = 0.038), whereas ubiquitin E3-encoding proteins (23 contigs) were expressed more strongly in genotype G2 (p-value = 0.096), suggesting that proteolysis occurred via different pathways in the two genotypes.

Analysis of the metabolic pathways for "T" contigs was limited because of the small number of contigs (155), which were distributed in several bins (Additional file 8). However, the expression of genes related to carbohydrate degradation and ethylene biosynthesis were found to be stronger for the NI treatment, whereas the expression of genes related to ribosomal protein synthesis and cell development appeared to be stronger for the IR treatment (Additional file 7).

**Figure 9:**
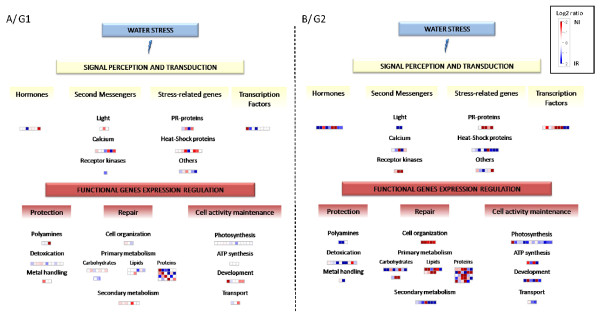
**Distribution of "GxT" contigs between metabolic pathways**. Each square represents the log_2_-transformed fold-change of abundance between irrigated (IR) and non-irrigated (NI) treatments for one contig: for genotype G1 (A) and G2 (B). Contigs in blue were overexpressed for the IR treatment and contigs in red were overexpressed for the NI treatment.

Different patterns were observed for "GxT" contigs (figure 9): i) Some pathways (25% of "GxT" contigs) displayed similar patterns in the two genotypes, but with responses of different magnitudes (i.e. scale plasticity, as defined by Lynch and Walsh [52]). Photosystem components tended to be overrepresented in genotype G2 in the IR condition, whereas few differences were observed between conditions for genotype G1. Conversely, genes related to cell organisation and PR-proteins were more likely to be overexpressed in the NI condition in genotype G2 compared to genotype G1, ii) Interestingly, most of the pathways (75% of "GxT"contigs) displayed opposite trends in the two genotypes (i.e. a re-ranking interaction effect [52]). For example, in the NI treatment, genes related to phenylpropanoid biosynthesis, auxin biosynthesis, heat stress, and light signalling were overexpressed in genotype G1, while they where underexpressed in genotype G2. Conversely, genes related to global primary metabolism (particularly starch degradation and ribosomal protein synthesis) and receptor kinases were overexpressed in genotype G2, but underexpressed in genotype G1 in the NI condition (additional file 7). This result strongly suggests that different metabolic pathways and genes were activated in these two genotypes in response to water shortage. Thus, these two genotypes exhibit different molecular strategies to cope with water deficit during the dry season.

## Discussion

### Gene discovery and expression analysis by NGS

Next-generation sequencing (NGS) technologies are becoming the method of choice for large-scale transcriptome analysis, even for non-model species (e.g. [29,41], reviewed in [53]). Several technologies have been developed, differing essentially in the number of reads generated and read length (reviewed in [54]), making it possible to catalogue the genes expressed and to monitor gene expression.

In *Eucalyptus*, Mizrachi *et al*. [38] generated 3.93 Gbp of short reads (36-60 bp) with sequencing-by-synthesis technology from Illumina, and assembled this information *de novo *into 18,894 contigs (Illumina-contigs) longer than 200 bp (22.1 Mbp in total). In this study, we obtained 0.398 Gbp of sequences with longer reads (mean of 350 bp), which were assembled into 48,950 contigs (454-sequencing contigs) with more than 200 bp each (36.5 Mbp in total). We were thus able to assemble more reads, i.e. 9.2% of the sequencing set, that is a much higher rate than the 0.56% reported by Mizrachi *et al*. [38]. BLAST searches for sequence similarities between the two datasets showed that 86% of the contigs were common to both studies (42,550 454-sequencing contigs matched 16,278 Illumina contigs). However, each Illumina contig matched a mean of five 454-sequencing contigs, indicating that UE detected with our approach were probably confounded in the short-read assembly. In addition, the short Illumina contigs may represent domains shared by multiple proteins, confirming the difficulty involved in assembling short reads into transcriptional units [55]. Alternatively we can not rule out the fact that genes were split in multiple contigs in the 454 assembly because of the lack of coverage compared to Illumina's short reads. Finally, we found that 36,985 454-UE did not match any previously described eucalyptus ESTs, and that 43% of these UE (18% of the 454-sequencing contigs) displayed no match with any nucleic acid or protein sequence published for any other species. Therefore, our 454-sequencing data considerably enriched the *Eucalyptus *EST collection.

RNA-Seq is also an interesting approach to obtain a comprehensive digital gene expression profile for specific tissues, cell types or developmental processes. In this study, the high degree of repeatability observed for the three replicates made it possible to test G, T and GxT effects with a statistical support. We were able to monitor the expression of 14,460 UE and to identify 1,445 UE displaying at least one significant effect. Some technical biases, such as non-linear amplification and a lack of sequencing depth may have resulted in a lack of precision in the prediction of gene expression by 454-sequencing (Additional file 9). Short read-based sequencing approaches provide ample read coverage and generally give better predictions of gene expression [56,57]. Thus, a combination of long and short reads may be seen as a reasonable strategy for the analysis of gene expression [55,58,59]. With this combined strategy, long-read sequencing can be used to establish a comprehensive catalogue of transcriptional units, while short reads mapped onto this assembly provide greater sequencing depth, improving predictions of abundance.

### Behaviour of variance components at the phenotypic and molecular levels

We observed that phenotypic and molecular variation are accounted for principally by genotypic differences. Indeed, above-ground biomass and contig abundance were influenced principally by genotype. Above-ground biomass was, on average, 49% higher for G2 than for G1, and most of the differentially expressed contigs (1,280 of 1,455) in this study presented a genotypic effect. The number of contigs overexpressed in one or the other of the genotypes was similar - 624 and 656 contigs were overexpressed in G1 and G2, respectively. No particular genotypic signature in terms of functional categories or pathways was detected. The two genotypes differed strongly in phenotype (not only it terms of growth potential, but also in terms of leaf morphology, stomatal distribution and water use efficiency), but it remains unclear whether these differences in transcript abundance were responsible for trait variation, neutral, or simply involved in reproductive isolation between parental species. Indeed, differences in gene expression between species have been reported in the field of ecological genomics [60,61] and interpreted as a mechanism of speciation. In our study, both genotypes were hybrid combinations between different species. This may have increased the number of differences between their transcriptomes. Further investigations about the role of gene expression in ecological speciation is an important question, particularly for eucalyptus, in which species complexes are common [62].

The variance accounted for by genotype-by-environment interaction (GEI) at the phenotypic and molecular level was also significant in this study. The NI treatment resulted in a significantly lower above-ground biomass, and this difference was greater for the least productive of the two genotypes, G1 (24%), than for G2 (12%). The G and T effects accounted for 56% and 13% of the above-ground biomass variation, respectively, whereas GxT effects accounted for only 0.2% of the variance. At the molecular level, we also found a higher proportion of genes displaying G effects (1,280 contigs, 8.8% of the contigs screened) but, surprisingly, we found fewer genes displaying T effects (155 contigs, 1.1% of the contigs screened) than GxT effects (274 contigs, 1.9% of the contigs screened).

While only 11 contigs were differentially expressed between the two treatments in genotype G1, 112 contigs showed differential expression in genotype G2 (4 contigs displayed differential expression for both genotypes). Moreover, when the whole *E. spp *sequencing set was screened, genotype G2 presented a larger number of specific contigs (10.5% of the *E.spp *sequencing set) than G1 (5.5% of the *E.spp *sequencing set). These results suggest that a larger set of genes is activated in genotype G2, leading to the triggering of specific responses to water deficit. This higher molecular sensitivity of genotype G2 may confer advantages ultimately resulting in a greater capacity to cope with water deficit during the dry season and, therefore, in stronger growth capacity (table 4).

**Table 4:** Summary of phenotypic and molecular plasticity evidenced for the two studied genotypes (G1 and G2), between irrigated (IR) and non irrigated (NI) treatments

		G1	G2
		**IR **→ **NI**	**IR **→ **NI**
**Above-ground biomass**		decrease	stable

**Transcript abundance**	Hormones	+	--
	Secondary messengers	+ (light signalling)	-- (light signalling) ++ (receptor kinases)
	Other stress related genes	+	-- (heat-shock proteins) ++ (PR proteins)
	Transcription factors	stable	++
	Cellular protection	+	--
	Damages repair	+ (carbohydrates secondary metabolism),	++ (cell organisation, lipids, proteins) -- (secondary metabolism)
	Cellular activity maintenance	stable	-- (photosynthesis, transport)

Abbreviations: +/- means that in a given category more than 50% of the genes are over/underexpressed in NI.++/-- means that in a given category more than 50% of the genes are over/underexpressed in NI with a log_2_-tranformed fold change <-0.8 or >0.8.

### Genes displaying GEI effects reflect differences in signal perception and response strategy

Contigs displaying GEI effects could be classified into two groups according to the function of the proteins encoded [63]: i) regulatory proteins responsible for drought signal transduction and response triggering, and ii) functional proteins involved in cell protection, damage repair and the maintenance of cell activity.

Regarding regulatory functions, the genes involved in the biosynthesis of hormones, such as ethylene and auxins in particular (aldo/keto reductase, proteins of the ethylene-responsive family, 2-oxoglutarate-dependent dioxygenase) were mostly overexpressed in genotype G1 and underexpressed in G2, in the NI condition. Genes acting as second messengers in the transduction of hormonal signal to stomatal guard cells [64,65] also displayed GEI effects: in the NI condition, genes involved in calcium signalling were predominantly overexpressed in G1, whereas the response of G2 to drought preferentially involved receptor kinases. We also identified other signal transducers, such as light-induced proteins and heat-shock proteins, which may be related to other types of stress induced by water deficit, including osmotic stress due to pH variations and oxidative stress due to the accumulation of reactive oxygen species (ROS) [66,67]. These pathways were mostly overexpressed in G1 and underexpressed in G2 during the dry season. Pathogenesis-related (PR) protein genes were overexpressed in G2, but displayed a less clear-cut pattern of expression in G1. PR proteins were initially reported to be induced by hormones or ROS in response to biotic stress [68,69], but they have also been shown to be involved in other abiotic stresses [70]. Lee *et al*. [69] also suggested that PR proteins may be used as storage proteins when growth is limited by environmental factors. Some transcription factors responded strongly to water shortage in genotype G2. Two, in particular, encoded factors homologous to *AtMYB12 *and *AtMYB85*, which have been shown to regulate secondary metabolism (flavonoid and lignin biosynthesis, respectively) in *Arabidopsis *[71]. These results suggest that water shortage induces different cellular stress cascades, perceived differently by the two genotypes.

Stress signal transducers interact to trigger the regulation of gene expression for the maintenance of three main functions: cell protection, damage repair and the maintenance of cell activity. Our results suggest that more genes related to cell protection were involved in the response to water shortage in genotype G1 than in genotype G2. Protection against drought stress seems to involve mostly carbohydrates, with 11 contigs displaying GEI effects, and, to a lesser extent, polyamines, which may modulate some ion channels [72].

By contrast to the trends observed for genes related to cell protection, more genes related to damage repair seem to be expressed during the dry season in genotype G2 than in genotype G1, particularly those related to cell organisation. The overexpression, during NI treatment, of genes related to primary metabolism, including carbohydrate, lipid and protein synthesis and degradation, suggests that resources are reallocated for the repair of cell structures or the formation of new structures in drought stressed plants. The patterns of expression of secondary metabolism genes differed between the two genotypes as well. As an example, G1 displayed a higher number of genes related to terpenoids and flavonoids synthesis (that may protect against oxidative stress) overexpressed in the NI treatment compared to genotype G2. However, the contrasts between NIR and IR treatments were much higher for G2. Conversely, genes related to lignin biosynthesis (e.g. *CCoAOMT*) were overexpressed in the IR condition only for G2.

Gene related to photosynthesis were found to be under-expressed in G2 subjected to NI treatment, whereas no variation was found in G1. Other metabolic processes, such cell development and transport, controlled by genes encoding water or sugar channels, decreased in NI treatment, particularly in G2. These results confirm the trends observed by Alexandersson *et al*. [73] in *Arabidopsis*. These authors studied the expression of 18 genes encoding aquaporins and showed that most of these genes were downregulated in leaves subjected to a gradual water deficit. Interestingly, cell activity seemed to be more reduced at the transcriptional level for G2, although this genotype grew more strongly. It is possible that, during water deficit, genotype G2 reduces its rate of photosynthesis and reallocates resources (as suggested by changes in primary metabolism) to preserve its cell structures and ability to resume growth when conditions become more favourable.

### Evolutionary implication behind GEI

We found that 31 of the 274 contigs displaying a GxT effect were absent from the G1 sequencing set (11.3%), whereas only two such contigs were absent in G2 (0.7%). Unfortunately, of 31 contigs absent in G1, 16 could not been assigned to a homolog gene in *Arabidopsis*. The others corresponded to genes related to cell organisation (ankyrins), ethylene synthesis, protein metabolism, PR proteins and receptor kinases. These genes may be considered non-essential for tree development, and are therefore unlikely to be subject to selection constraints. Landry *et al*. [74] found an overrepresentation of non-essential genes (the deletion of which is not lethal) among genes displaying GEI in *Saccharomyces cerevisiae*. They proposed two hypotheses to account for the activity of these genes being compensated in cells: i) metabolic buffering: non-coded metabolites may be rerouted through the metabolic network, and ii) genetic buffering: paralogous genes may supply the missing function. We showed in the results section (Additional file 5) that differences between genotypes may be accounted for by the preferential expression of different members or splicing forms of genes from the same family. This observation may confirm the hypothesis of genetic buffering.

Scale plasticity was observed for 146 of the 274 differentially expressed contigs: genotype ranks were conserved between treatments but one genotype reacted more or less strongly to the environmental variation. Conversely, 90 contigs showed a change in ranking between genotypes (rank plasticity). Landry *et al*. [74] hypothesised that these two types of GEI would have different effects on the evolution of plastic traits. In the case of scale plasticity, whatever the environment, selection would result in the same favoured genotype, whereas in the case of rank plasticity, different genotypes would be selected in different environments. In the present study, we found differentially expressed genes presenting both scale plasticity (62%) and rank plasticity (38%), indicating different types of reaction norms on which natural selection would act on.

## Conclusions

We showed that next-generation sequencing is a powerful tool for transcriptome screening: with 398 Mb of sequence, we were able to assemble ESTs into 69,584 contigs, with remaining 80,245 singletons, and to determine the relative abundance of 14,460 contigs each comprising more than 10 reads. Large differences between genotypes, in terms of phenotypic behaviour and transcriptome regulation, were observable. Differences in gene expression between the two genotypes appear to affect the whole transcriptome, rather than specific pathways. The genotype-specific response to water shortage (i.e. GxT effect) was more pronounced than the response common to both genotypes (i.e. T effect). The genes displaying genetically controlled plasticity were found to belong to a number of different pathways essentially related to signal transduction and primary metabolism. The more productive genotype, G2, express a larger set of genes, leading to the triggering of specific molecular responses. Moreover, GxT interaction results principally from a lack of molecular response in genotype G1, together with a strong response of genotype G2 (table 4). The ability to regulate more actively its transcriptome might be a key component in the maintenance of biomass in water deficit conditions.

Finally, although this study provides clues to the way in which different genotypes activate their transcriptomes when subjected to water deficit, more research is required to understand the molecular mechanisms involved during the dry season. First, there is a need to characterize reaction norm in a broader genetic background [75]. Second, epigenetics or post-transcriptional regulation mechanisms that are well known to interfere with abiotic stress responses [76,77] deserve specific investigations.

## Authors' contributions

EV: participated in the field work, performed the molecular work, carried out the statistical analysis and drafted the manuscript. CK and CN performed the bioinformatic work (assembly and annotation). EN and MK provided *E. grandis *sequencing sets and participated in the statistical analysis. CP participated in co-ordination of the molecular, bioinformatic and statistical work and in drafting the manuscript. JMG participated in the design of the study, co-ordination of the field, molecular, bioinformatic and statistical work and drafting the manuscript. All authors read and approved the final version of the manuscript.

## Supplementary Material

Additional file 1 **Results of the ANOVA for above-ground biomass**. Click here for file

Additional file 2 **Distribution of read length for the three half-runs**. Click here for file

Additional file 3 **Increasing coverage with successive runs**. Number of contigs represented in each half-run or combination of several half-runs. Performing a second half-run increased contig coverage by an average of 18%, and a third half run increased coverage by an average of 6%. Click here for file

Additional file 4 **Comparison of the distribution of Gene Ontology (GO) categories between *Eucalyptus spp *unigene elements (UE) and *Arabidopsis *annotated unigenes**. Proportion of each GO category (Biological Process, Cellular Component and Molecular Function) found in the *E. spp *sequencing set and in the annotated *Arabidopsis *genome. Click here for file

Additional file 5 **Differentially expressed contigs displaying significant T and GxT effects**. Each contig showing T or GxT is associated with a functional category (as defined by Mercator : http://mapman.gabipd.org/web/guest). Log_2_-transformed fold change between abundance in irrigated and non irrigated libraries are indicated for each genotype, as well as p-value of T and GxT effects analyzed by the ANOVA. Click here for file

Additional file 6 **Distribution of "G" contigs between functional pathways**. Each square represents the log_2_-transformed fold-change of abundance between genotypes 1-41 and 18-50 for one contig. Contigs in green were overexpressed in genotype 1-41 and contigs in blue were overexpressed in genotype 18-50. Click here for file

Additional file 7 **Significant categories for the Wilcoxon rank sum test, according to Mapman analysis for the pairwise comparison of differentially expressed contigs displaying genotype (G), treatment (T) and genotype × treatment (GxT) effects**. ** Categories differentially expressed at an error rate threshold of 0.05 * Categories differentially expressed at an error rate threshold of 0.1 Click here for file

Additional file 8 **Distribution of "T" contigs between several functional pathways**. Each square represents the log_2_-transformed fold-change of abundance between irrigated (IR) and non-irrigated (NI) treatments for one contig. Contigs in blue were overexpressed for the IR treatment and contigs in red were overexpressed for the NI treatment. Click here for file

Additional file 9 **Supporting information: validation of digital profiles by analyzing expression by RT-qPCR on 36 genes**. Click here for file
