# Supplementary material for: RNA-Seq reveals genotype-specific molecular responses to water deficit in eucalyptus
Source: BMC Genomics. 2011 Nov 2;12:538. doi: 10.1186/1471-2164-12-538 (PMC3248028; doi:10.1186/1471-2164-12-538)
Supplement: Additional file 1 — Results of the ANOVA for above-ground biomass. [file 1471-2164-12-538-S1.PDF]

Source	DDL	Sum of squares	Mean of squares	F	Pr > F
Genotype	1	183.059	183.059	75.868	< 0.0001
Treatment	1	43.192	43.192	17.901	0.0001
Genotype x Treatment	1	0.934	0.934	0.387	0.537
