# Supplementary figures and images for: RNA-Seq reveals genotype-specific molecular responses to water deficit in eucalyptus
Source: BMC Genomics. 2011 Nov 2;12:538. doi: 10.1186/1471-2164-12-538 (PMC3248028; doi:10.1186/1471-2164-12-538)

# RUN A

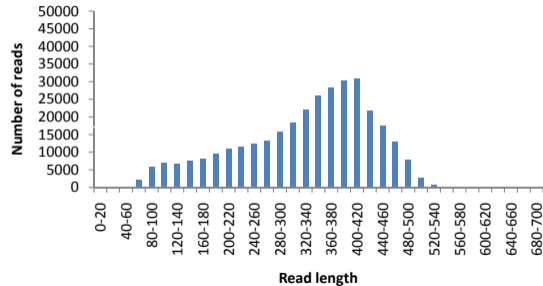

# RUN B

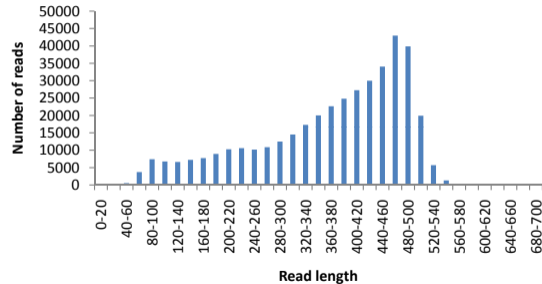

# RUN C

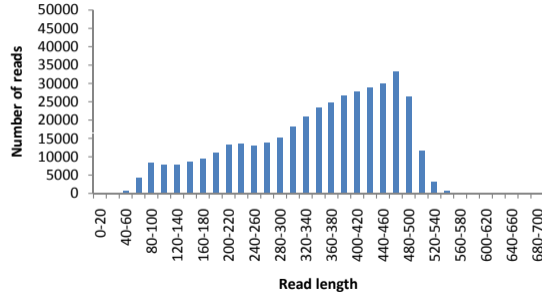

Supplement: Additional file 2 — Distribution of read length for the three half-runs. [file 1471-2164-12-538-S2.PDF]

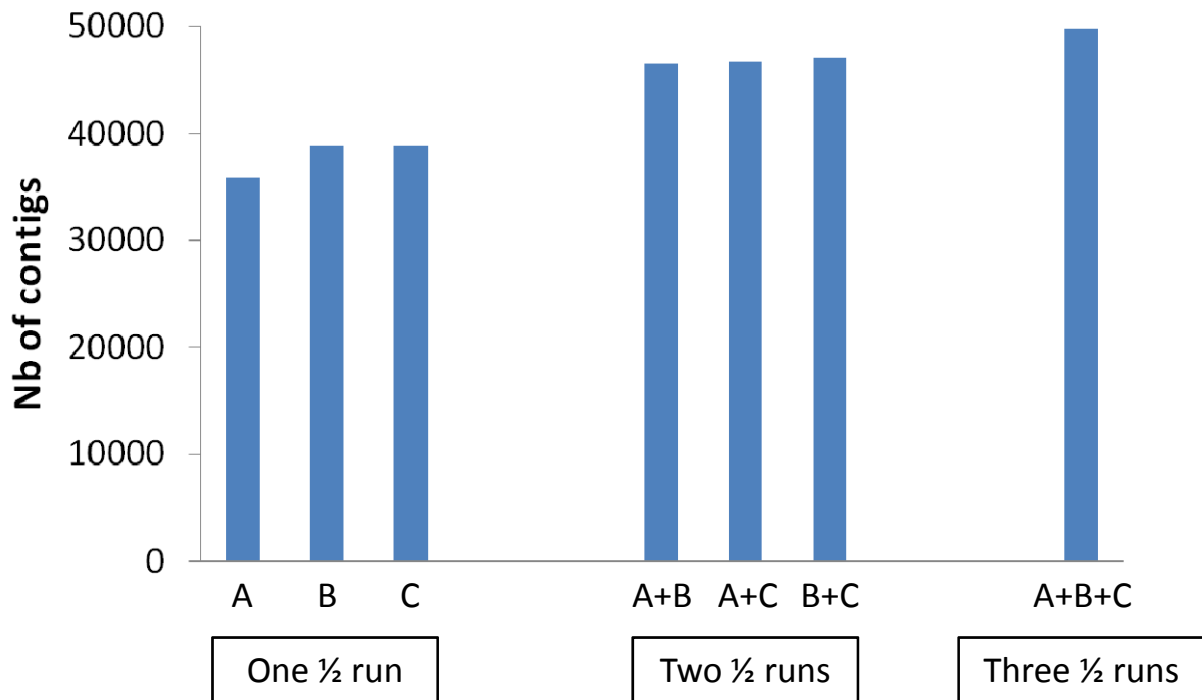

Supplement: Additional file 3 — Increasing coverage with successive runs. Number of contigs represented in each half-run or combination of several half-runs. Performing a second half-run increased contig coverage by an average of 18%, and a third half run increased coverage by an average of 6%. [file 1471-2164-12-538-S3.PDF]

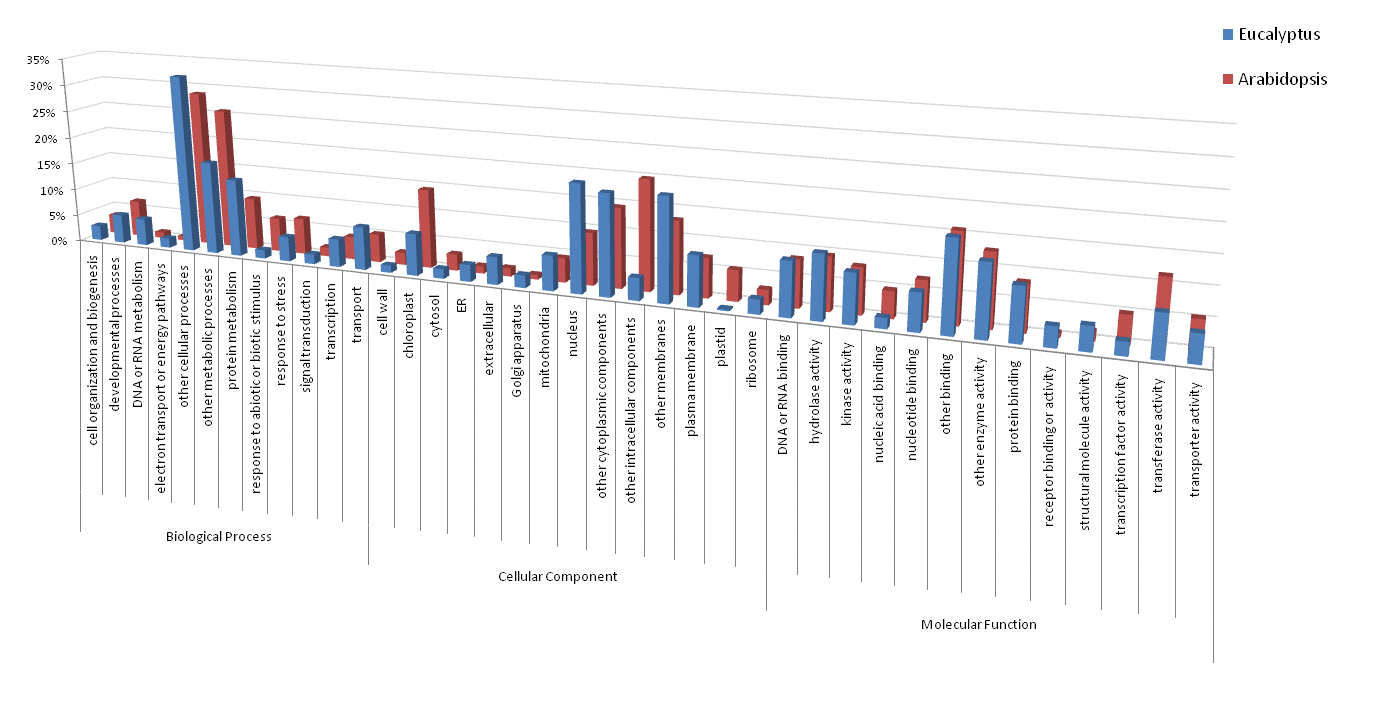

Supplement: Additional file 4 — Comparison of the distribution of Gene Ontology (GO) categories between Eucalyptus spp unigene elements (UE) and Arabidopsis annotated unigenes. Proportion of each GO category (Biological Process, Cellular Component and Molecular Function) found in the E. spp sequencing set and in the annotated Arabidopsis genome. [file 1471-2164-12-538-S4.GIF]
