# Supplementary material for: RNA-Seq reveals genotype-specific molecular responses to water deficit in eucalyptus
Source: BMC Genomics. 2011 Nov 2;12:538. doi: 10.1186/1471-2164-12-538 (PMC3248028; doi:10.1186/1471-2164-12-538)
Supplement: Additional file 6 — Distribution of "G" contigs between functional pathways. Each square represents the log2-transformed fold-change of abundance between genotypes 1-41 and 18-50 for one contig. Contigs in green were overexpressed in genotype 1-41 and contigs in blue were overexpressed in genotype 18-50. [file 1471-2164-12-538-S6.PDF]

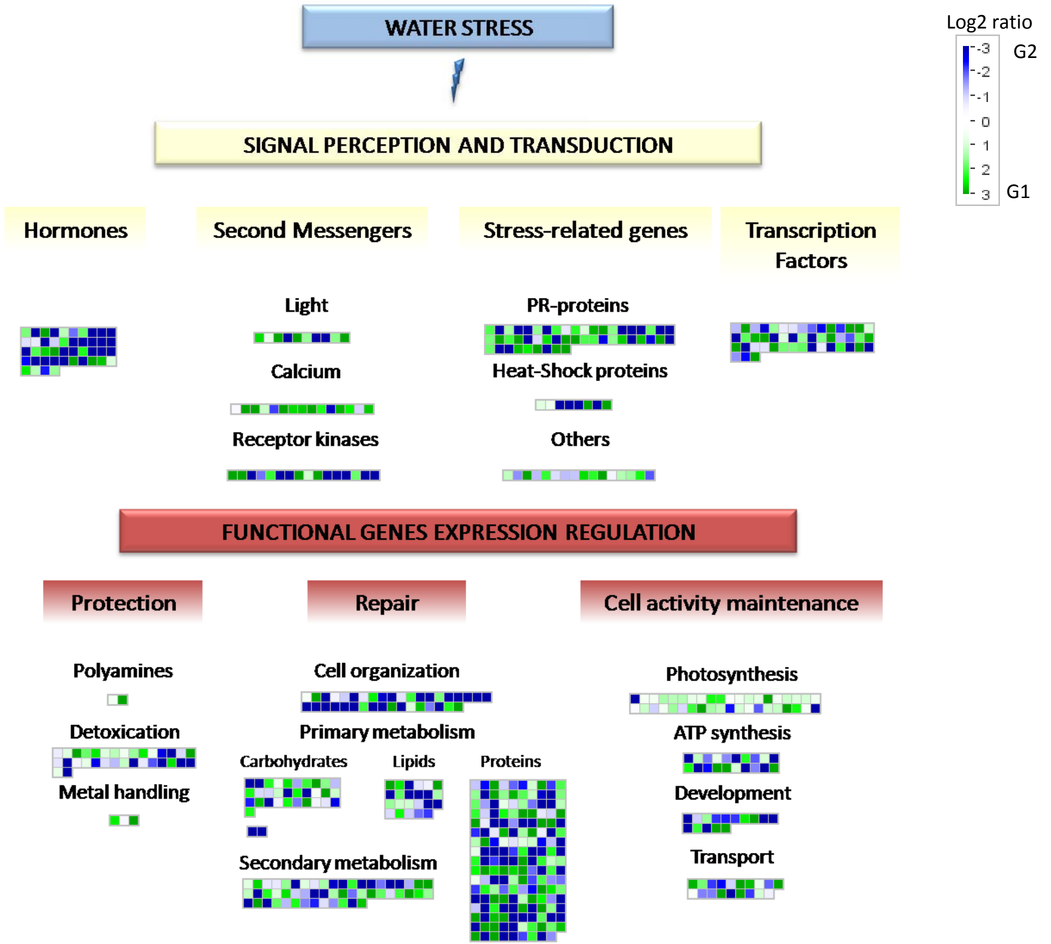
WATER STRESS
SIGNAL PERCEPTION AND TRANSDUCTION
Hormones
Second Messengers
Stress-related genes
Transcription Factors
Light
Calcium
Receptor kinases
PR-proteins
Heat-Shock proteins
Others
FUNCTIONAL GENES EXPRESSION REGULATION
Protection
Repair
Cell activity maintenance
Polyamines
Detoxication
Metal handling
Cell organization
Primary metabolism
Carbohydrates
Lipids
Proteins
Secondary metabolism
Photosynthesis
ATP synthesis
Development
Transport
Log2 ratio
-3
-2
-1
0
1
2
3
G2
G1
