# Supplementary material for: RNA-Seq reveals genotype-specific molecular responses to water deficit in eucalyptus
Source: BMC Genomics. 2011 Nov 2;12:538. doi: 10.1186/1471-2164-12-538 (PMC3248028; doi:10.1186/1471-2164-12-538)
Supplement: Additional file 7 — Significant categories for the Wilcoxon rank sum test, according to Mapman analysis for the pairwise comparison of differentially expressed contigs displaying genotype (G), treatment (T) and genotype × treatment (GxT) effects. ** Categories differentially expressed at an error rate threshold of 0.05 * Categories differentially expressed at an error rate threshold of 0.1 [file 1471-2164-12-538-S7.PDF]

Category	"G" contigs: (1-41 vs 18-50)		"T"contigs: (IR vs NI)		"GxT"contigs: 1-41 (IR vs NI)		"GxT"contigs: 18-50 (IR vs NI)		"GxT"contigs: IR (1-41 vs 18-50)		"GxT"contigs: NI (1-41 vs 18-50)	
	# contigs	mean FC	# contigs	mean FC	# contigs	mean FC	# contigs	mean FC	# contigs	mean FC	# contigs	mean FC
hormone metabolism	44	-2**	5	-0.77	7	-0.14	9	0.25	9	-1.42*	10	-0.51
hormone metabolism : auxin	8	-1.22	1	-1.53	2	-0.21	2	2.67*	2	-1.44	2	1.45
hormone metabolism : ethylene signal transduction	21	-3.22**	1	-6.36*	0	NA	3	-2.37	2	-5.24**	3	-5.39**
signalling : calcium	14	1.89**	0	NA	6	-0.48	4	0.57	6	0.87	6	2.56*
signalling : light	10	0.15	5	0.11	3	-0.47	2	2.88*	3	0.27	3	3.52*
signalling : receptor kinases	16	-1.07	1	1.09	1	1.25	3	-2.74*	1	-0.89	3	-4*
stress	73	-0.05	13	-0.68	17	-0.27	19	0.22	20	-0.81**	21	-0.21
abiotic stress : heat	10	-0.85	6	-0.26	9	-0.7*	10	1.48*	10	-1.34**	10	0.68
biotic stress : PR-proteins	29	0.51**	3	-1.63	3	-0.2	3	-2.76*	4	-0.4	5	-0.88
regulation of transcription : MYB-related transcription factor family	3	0.52	2	1.5*	0	NA	0	NA	0	NA	0	NA
regulation of transcription : unclassified	6	-2.15	2	-0.04	1	0.16	1	7.42*	1	1.25	1	8.51*
nitrilases	7	2.12*	1	0.97	0	NA	0	NA	0	NA	0	NA
photosystem	38	0.94*	10	0.73	13	0.1	13	0.99*	13	0.38	13	1.28
cell	37	-2.31**	5	-1.62	3	0.03	5	-2.7**	5	-1.37	6	-2.91**
major CHO metabolism : starch degradation	2	-3.67	3	-2.76**	1	0.56	2	-2.16*	1	-0.79	2	-2.67*
lipid metabolism	14	-0.59	1	-5.07	5	0.25	6	-1.47	5	1.19*	6	-0.49
protein degradation : serine protease	9	1.75**	0	NA	0	NA	1	-2.61	1	0.05	1	-4.43
protein degradation : ubiquitin E3	23	-2.04*	3	-1.99	5	-0.39	6	-0.4	5	0.02	6	-0.52
protein synthesis : ribosomal protein	47	-0.47	3	1.26*	2	1.93**	3	-1.6	3	-0.53	3	-3.72*
secondary metabolism : phenylpropanoids	24	0.11	2	0.84	4	-0.24	3	2.97**	4	1.7**	4	4.16**
development	14	-0.62	2	1.58*	6	-0.63	6	0.64	6	0.42	7	1.32

**Categories differentially expressed ay an error threshold of 0.05

*Categories differentially expressed ay an error threshold of 0.1
