# Supplementary material for: RNA-Seq reveals genotype-specific molecular responses to water deficit in eucalyptus
Source: BMC Genomics. 2011 Nov 2;12:538. doi: 10.1186/1471-2164-12-538 (PMC3248028; doi:10.1186/1471-2164-12-538)
Supplement: Additional file 8 — Distribution of "T" contigs between several functional pathways. Each square represents the log2-transformed fold-change of abundance between irrigated (IR) and non-irrigated (NI) treatments for one contig. Contigs in blue were overexpressed for the IR treatment and contigs in red were overexpressed for the NI treatment. [file 1471-2164-12-538-S8.PDF]

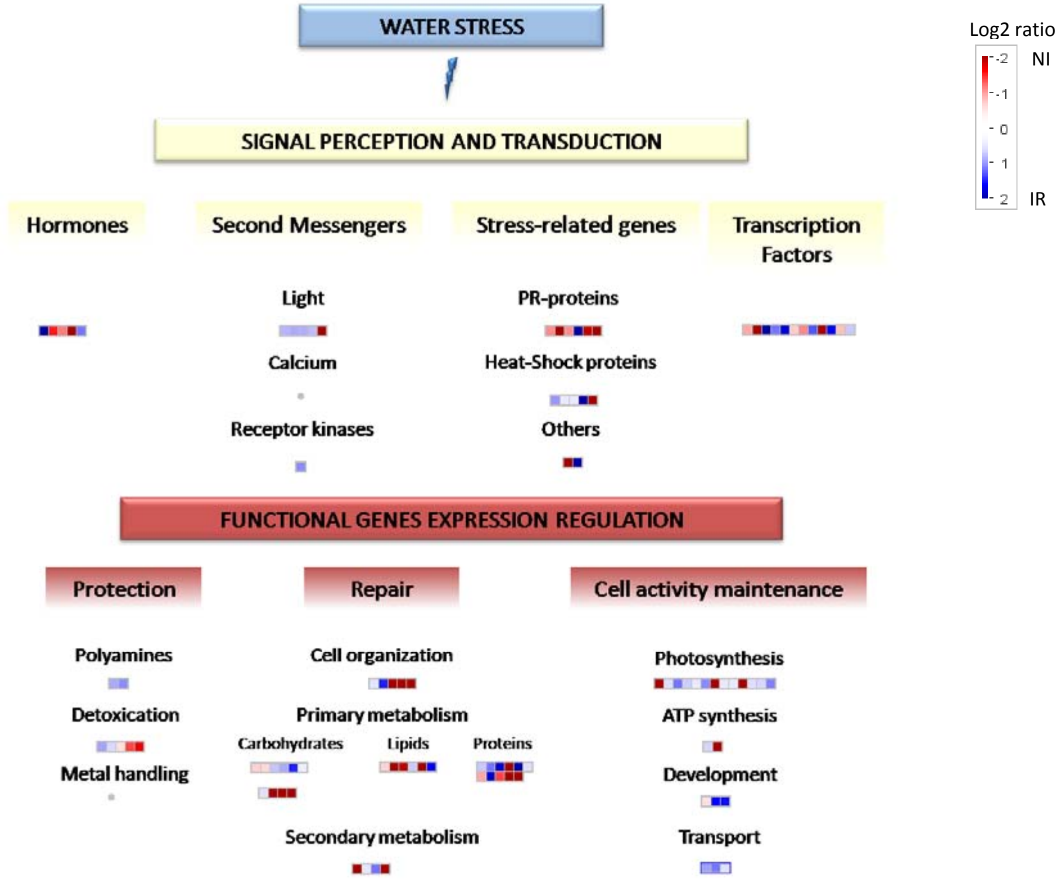
WATER STRESS
SIGNAL PERCEPTION AND TRANSDUCTION
Hormones
Second Messengers
Stress-related genes
Transcription Factors
Light
PR-proteins
Calcium
Heat-Shock proteins
Receptor kinases
Others
FUNCTIONAL GENES EXPRESSION REGULATION
Protection
Repair
Cell activity maintenance
Polyamines
Cell organization
Photosynthesis
Detoxication
Primary metabolism
ATP synthesis
Carbohydrates
Lipids
Proteins
Metal handling
Development
Secondary metabolism
Transport
Log2 ratio
-2
-1
0
1
2
NI
IR
