# Supplementary material for: RNA-Seq reveals genotype-specific molecular responses to water deficit in eucalyptus
Source: BMC Genomics. 2011 Nov 2;12:538. doi: 10.1186/1471-2164-12-538 (PMC3248028; doi:10.1186/1471-2164-12-538)
Supplement: Additional file 9 — Supporting information: validation of digital profiles by analyzing expression by RT-qPCR on 36 genes. [file 1471-2164-12-538-S9.PDF]

Validation by RT-qPCR

Methods

The transcript abundance of 36 genes was estimated by RT-qPCR for three types of sample: i) cDNA samples (obtained with the Smart cDNA Library Construction kit) used for sequencing, ii) RNAs used for library construction and sequencing, and iii) RNAs extracted from independent samples. The extracted RNAs obtained in (ii) and (iii) were reverse-transcribed with the ImpromII Reverse Transcription System (Promega, Madison, WI, USA) according to the manufacturer's instructions. Primer pairs were designed from the 454-reads with Primer3 software (Rozen and Skaletsky, 2000) (see supporting file 1). Real-time PCR was performed on 384-well plates, and was monitored with a Light Cycler 480 (Roche Applied Science, Indianapolis, IN, USA) at the Genome and Transcriptome Facility of Bordeaux (France, *https://www4.bordeaux-aquitaine.inra.fr/pgtb),* France. Each reaction mixture contained 3 µl of cDNA preparation (diluted 1/50 for Improm reverse-transcribed cDNA and 1/1000 for SMART reverse-transcribed cDNA), 5 µl of 2 x ABsolute QPCR SYBR Green Mix (ABgene, Epsom, UK) and 70 nM gene-specific primer in a total volume of 10 µl. The cDNA samples and master mix were pipetted with a Hamilton workstation (Hamilton Robotics, Reno, NV, USA). Each reaction was performed in duplicate. The fluorescent signal was evaluated and starting concentrations were calculated with LinRegPCR (Ramakers *et al.* 2003; Ruijter *et al.* 2009). The relative abundance of transcripts was normalised against the mRNA levels for four constitutively expressed housekeeping genes: elongation factor 1-α (EF1-α), elongation factor 2 (eEF2), glyceraldehyde 3P dehydrogenase (G3PDH) and cyclophilin (Cyp), by calculating a normalisation factor with GeNorm (Pattyn *et al.*, 2003). Log2-transformed ratios between treatments were generated for each genotype, to facilitate comparison of the results with those of digital expression profiling.

Results

We selected a subset of 36 contigs for validation of their expression levels by RT-qPCR. Sixteen displayed stable levels of expression (0.5<FC<2), and 21 showed a contrasted expression pattern (FC<0.5 or FC>2) in the digital expression analysis, between genotypes or treatments, or displaying GxT effects. Four stably expressed genes were chosen with GeNorm for data normalisation: these genes encoded elongation factor 1-alpha (*EF1a*), eukaryotic translation elongation factor 2 (*eEF2*), cyclophilin (*CYP*), and glycerol-3-phosphate dehydrogenase (*G3PDH*). RT-qPCR was carried out on three sets of RNA/cDNA samples: i) 8 cDNA samples (used for 454-sequencing) obtained by SMART amplification of eight RNA samples corresponding to the four conditions with two replicates (set #1), ii) eight new cDNA samples obtained by linear reverse transcription (RT) of the same RNA samples (set #2), iii) eight new cDNA samples obtained by RT of eight new RNA samples (set #3).

Gene expression, as analysed by RT-qPCR, was strongly correlated in sets #2 and #3 (significant Spearman correlation of 93.4%), suggesting a high degree of homogeneity in the pattern of expression between biological replicates. Correlations between gene expression in these two sets and in set #1 were weaker, with a mean correlation of 68.3%. The non-linear SMART amplification may account for these differences in expression patterns and suggests that caution is required when using this technique for expression validation. We therefore chose not to use this method in subsequent analyses. Spearman's correlations between digital expression profiles (454-results) and the two sets of RT-qPCR results were significant, but relatively low: 35.3% (set #2), 28.7% (set #3). The weakness of these correlations may be accounted for by the successive biases of the method: non-linear amplification by SMART and low sequencing depth, generating only a limited number of reads for digital expression analysis. Nevertheless, DEGseq analysis of the 11 contigs with significant fold-changes

between IR and NI treatments for the two genotypes in 454-sequencing data, confirmed the expression patterns of seven genes in set #1, ten genes in set #2, and seven genes in set #3 (supporting file 2). The reaction norms of molecular variations cannot be fully described by 454 quantification (Spearman's correlations between the results obtained with the two methods vary from 28.7% to 35.3%), the pattern of molecular plasticity of significant contigs was similar for the different types of expression quantification.

Supporting file 1 – Primers used for validation by RT-qPCR

Gene Code	Gene Name	Contig Name	Primer Forward (5'-3')	Primer Reverse (5'-3')
SHINE1	DNA binding / transcription factor	F0F0A3T01A00IG.l.eu.2	TCAAGAAATGCAGCAAGACG	GACCCAGTTGGAGTCTGAGC
LP1	nonspecific lipid transfer protein 1	F0F0A3T01A00NX.l.eu.2	AGGGGATCGACTTCAACCTC	GAGCTCGTGGACCTCACTTC
SIP2	hydrolase, hydrolyzing O-glycosyl compounds	F0F0A3T01A032B.l.eu.2	CGATTTGGCGCTTACTCTTC	ACCGGCCATCTGTACATCTC
LIM1	transcription factor	F0F0A3T01A03RQ.l.eu.2	AGAGGGAAACAATGGCTCCT	GCTTACAACCAACCCGAGAA
RD26	NAC domain containing protein 2; transcription factor	F0F0A3T01A0DJA.l.eu.2	CAACACACCTCCGCAATATG	TCCCAACCTTCCAACTTCAC
DCAM	adenosylmethionine decarboxylase family protein	F0F0A3T01A0ESR.l.eu.2	ATCCAGAGGGTGTTGGTCTG	CTCCAGCACTGTACCCCTTC
CHIAB	basic chitinase	F0F0A3T01A0IP5.l.eu.2; F0F0A3T01A3DVE.l.eu.2	CAAGGGCTTCTACACTTACG	AGCAGTATCCCCACGCATAG
ELIP1	(EARLY LIGHT-INDUCABLE PROTEIN); chlorophyll binding	F0F0A3T01A0S92.l.eu.2	TCCCTCTTCATCGCCTACAC	GTCTGCCATTGATCCTCTCC
PHS2	ALPHA-GLUCAN PHOSPHORYLASE 2	F0F0A3T01A0SF1.l.eu.2	TTCTAGCTTCCCTCCCTTCC	GTTAGGCGGAGTGCATTTGT
CCoAOMT2	caffeoyl-CoA 3-O-methyltransferase, putative	F0F0A3T01A0Z5S.l.eu.2; F0F0A3T01BLVGJ.l.eu.2	GCAAGATGAGAAGAACCATG	AGCACGAAGTCCCGGTAGTA
UCR	ubiquinol-cytochrome C reductase complex, putative	F0F0A3T01A1SQD.l.eu.2	GCTGTCTCCTGACGTTTGTG	CCGGCTATTTCGACTCCTTC
DELTA-TIP	delta tonoplast integral protein	F0F0A3T01A2Q8I.l.eu.2	CCGTGGTCAGTGGAGACTTC	CATTGACAAGAGGTGCATGG
CesA1	CELLULOSE SYNTHASE 1	F0F0A3T01A31VD.l.eu.2	TAAAGTGCTTGCTGGCATTG	TGAGAAGCGATGTCCATTTG
PIP1B	plasma membrane intrinsic protein 1;2	F0F0A3T01A344C.l.eu.2	TTCTGCAACATGGCTACACC	ATTCCTCTTGGCATCAGTGG
CCoAOMT1	caffeoyl-CoA 3-O-methyltransferase, putative	F0F0A3T01A4G7A.l.eu.2	GTATGTGAGGTACTACAGGG	CTCTCTTTTTCGTGGGTTCT
LHY1-2	LATE ELONGATED HYPOCOTYL	F0F0A3T01A4GAO.l.eu.2	GAAAGAGGATAATGGGCCAAG	CCTCGGGAAATTATGAGCAG
RBCL	large subunit of RUBISCO	F0F0A3T01A5ANA.l.eu.2	GCTAAGAACTACGGTAGAGC	TTCACCTGTTTCAGCCTGTG
ERD15	EARLY RESPONSIVE TO DEHYDRATION 15	F0F0A3T01A5C8L.l.eu.2	ATATTTCGCCGTCGTTCAAG	ATAGAGGGGCATCTGGGTTC
PGR5	PROTON GRADIENT REGULATION 5	F0F0A3T01A5OGX.l.eu.2 ; F0F0A3T01A1FPP.l.eu.2	TGGCTTGTTGCTAGGGTTTC	ATTGATCGCGAGCACTTTTC
GDR1	GLUTATHIONE-DISULFIDE REDUCTASE	F0F0A3T01A5Q34.l.eu.2	TAAGTGTGGTGCAACGAAGG	AACCCGTCTTGTGACTGACC
2-ODD	2-oxoglutarate-dependent dioxygenase	F0F0A3T01A8608.l.eu.2	CTCCAGATCATGTCCAACGA	CAAACAGGCTTTCACAATCG
HYP1	HYPOTHETICAL PROTEIN 1	F0F0A3T01A8IGK.l.eu.2	CCGGTGATGTGAGAGAAACC	AGCGGTATACGCGACTTGAG
RPP1A	60S acidic ribosomal protein P1	F0F0A3T01AFPEY.l.eu.2	CACCAATGTTGGTTCTGGTG	AGACCAAAGCCCATGTCATC
GHP3	glycosyl hydrolase family 3 protein	F0F0A3T01AJF18.l.eu.2	GGGTTGCAGACGTTCTGTTT	ATGAGGATCCCCAACATTCA
DXR	1-DEOXY-D-XYLULOSE 5-PHOSPHATE REDUCTOISOMERASE	F0F0A3T01AJOYE.l.eu.2	TGAGATCATTCCAGGGGAAC	GCTATGTCCTTGCCTGCTTC
HEN4	HUA ENHANCER 4; nucleic acid binding	F0F0A3T01AYA64.l.eu.2	GAGAGGAAGGTGGATGCTTG	TGAGCTGCATGAGTTTGCTC
HVA22	abscisic acid-responsive HVA22 family protein	F0F0A3T01B0RI8.l.eu.2	TCGAGAGCAAATCACCAATG	CTCTCACCGTCTGCACTCTG
LHY1-1	LATE ELONGATED HYPOCOTYL	F0F0A3T01B9JR1.l.eu.2	CGTTGAAAGAGGGAGTTCAGG	GAGCAGTTTGCATCTCATCTG
POLG	polygalacturonase, putative	F0F0A3T01BFXHG.l.eu.2	CGAAACCAAGATGTGTGTGG	CGATCAAGACCTCCTGCTTC
CER6	CUTICULAR 1; catalytic	F0F0A3T01EOHZ5.l.eu.2	TCCATATTCCCGAAGTGGTC	CGCAATCCACCTATCAAACC

Supporting file 2 – Comparison of fold-change (FC) ratio between RT-qPCR and digital analysis for 30 genes. Expression assessed by determining the amount of cDNA obtained with the SMART construction kit (Set #1) and in two biological replicates (Set #2 and Set #3) by linear reverse transcription (RT) was compared with the results for 454-sequencing. The mean log2-transformed FC values for the two biological replicates are indicated

				Log2 FC : [IR/NI]								Fold Change Similarity		
				454		Set 1 (SMART)		Set 2 (RT)		Set 3 (RT)				
**Gene Code**	**Gene Name**	**Contig Name**	**nb reads**	**1-41**	**18-50**	**1-41**	**18-50**	**1-41**	**18-50**	**1-41**	**18-50**	**Set #1/ 454**	**Set #2/ 454**	**Set #3/ 454**
LP1	nonspecific lipid transfer protein 1	F0F0A3T01A00NX.l.eu.2	2751	1.42*	-1.6*	1.36	-0.6	3.12	n.a. (-)[c]	n.a. (+)	-2.58	Y	Y	Y
ELIP1	(EARLY LIGHT-INDUCABLE PROTEIN); chlorophyll binding	F0F0A3T01A0S92.l.eu.2	317	-0.07	3.32*	-0.77	-0.34	n.a. (+)[b]	0.02	4.32	0.51	N	Y	Y
SIP2	hydrolase, hydrolyzing O-glycosyl compounds	F0F0A3T01A032B.l.eu.2	463	0	1.98*	-1.13	-0.81	2.89	2.35	4.02	-0.64	N	Y	N
LHY1-2	LATE ELONGATED HYPOCOTYL	F0F0A3T01A4GAO.l.eu.2	275	0.62	4*	2.19	3.13	4.46	0.62	4.17	2.15	Y	Y	Y
DCAM	adenosylmethionine decarboxylase family protein	F0F0A3T01A0ESR.l.eu.2	392	0.34	1.88*	0.85	1.59	3.6	0.26	3.22	1.3	Y	Y	Y
DELTA-TIP	delta tonoplast integral protein	F0F0A3T01A2Q8I.l.eu.2	637	0.02	1.24*	-0.02	2.21	4.12	2.28	2.39	2.26	Y	Y	Y
LHY1-1	LATE ELONGATED HYPOCOTYL	F0F0A3T01B9JR1.l.eu.2	75	0.77	4.93*	1.89	0.09	0.38	0.52	0.49	0.21	Y	Y	Y
PIP1B	plasma membrane intrinsic protein 1;2	F0F0A3T01A344C.l.eu.2	177	0.06	4.43*	1.19	1.1	2.13	-2.05	n.a. (+)	n.a. (-)	Y	N	N
PHS2	ALPHA-GLUCAN PHOSPHORYLASE 2	F0F0A3T01A0SF1.l.eu.2	43	0.87	-2.53*	-0.35	-0.4	0.35	-0.68	-0.22	-0.61	N	Y	N
PGR5	PROTON GRADIENT REGULATION 5	F0F0A3T01A5OGX.l.eu.2	209	0.49	2.14*	1.25	3.45	2.26	2.59	2.14	3.7	Y	Y	Y
CHIAB	basic chitinase	F0F0A3T01A0IP5.l.eu.2	290	-1.11*	-1.07*	-1.41	-1.12	-7.5	-5.71	-6.97	-10.93	Y	Y	Y
UCR	ubiquinol-cytochrome C reductase complex, putative	F0F0A3T01A1SQD.l.eu.2	303	0.45	1.04	0.23	0.21	n.a. (+)	0.83	2.88	-0.08	Y	Y	N
HEN4	HUA ENHANCER 4; nucleic acid binding	F0F0A3T01AYA64.l.eu.2	22	-0.13	-3.48	0.65	-0.82	2.24	-0.34	8.99	-0.17	Y	N	N
POLG	polygalacturonase, putative	F0F0A3T01BFXHG.l.eu.2	21	n.a.[a]	-2.11	-0.51	-1.8	n.a.	-2.02	n.a.	-1.89	Y	Y	Y
SHINE1	DNA binding / transcription factor	F0F0A3T01A00IG.l.eu.2	205	-0.07	-0.65	-0.14	0.55	n.a.	-3.69	n.a.	-2.12	N	Y	Y
2-ODD	2-oxoglutarate-dependent dioxygenase	F0F0A3T01A8608.l.eu.2	46	1.99	-1.48	0.75	0.68	2.99	-2.05	3.34	-2.88	N	Y	Y
HYP1	HYPOTHETICAL PROTEIN 1	F0F0A3T01A8IGK.l.eu.2	23	-0.54	-2.14	0.78	0.59	5.26	0.33	4.7	2.11	N	N	N
HVA22	abscisic acid-responsive HVA22 family protein	F0F0A3T01B0RI8.l.eu.2	18	3.19	3.78	-0.71	-0.91	4.35	-6.26	4.54	-1.97	N	N	N
DXR	1-DEOXY-D-XYLULOSE 5-PHOSPHATE REDUCTOISOMERASE	F0F0A3T01AJOYE.l.eu.2	68	1.31	1.26	0.94	4.21	2.45	1.14	2.95	3.62	Y	Y	Y
GHP3	glycosyl hydrolase family 3 protein	F0F0A3T01AJF18.l.eu.2	55	0.46	-1.15	1.45	-1.48	1.65	-0.59	0.93	-1.42	Y	Y	Y
ERD15	EARLY RESPONSIVE TO DEHYDRATION 15	F0F0A3T01A5C8L.l.eu.2	162	-0.13	0.59	-0.23	-0.1	2.84	0.19	0.66	0.36	N	N	N
RPP1A	60S acidic ribosomal protein P1	F0F0A3T01AFPEY.l.eu.2	15	-3.3	-2.03	-0.59	0.08	1.73	1.94	-0.55	0.26	N	N	N
LIM1	transcription factor	F0F0A3T01A03RQ.l.eu.2	156	-0.99	-0.4	-4.92	1.9	n.a.	-2.24	n.a.	-3.06	N	Y	Y
RD26	NAC domain containing protein 2; transcription factor	F0F0A3T01A0DJA.l.eu.2	51	-0.13	0.88	0.94	3.98	3.01	-1.01	1.79	n.a.	N	N	Y
CCoAOMT2	caffeoyl-CoA 3-O-methyltransferase, putative	F0F0A3T01A0Z5S.l.eu.2	114	-0.57	0.68	-0.12	0.52	-0.04	0.35	-0.04	-0.27	Y	Y	N
GDR1	GLUTATHIONE-DISULFIDE REDUCTASE	F0F0A3T01A5Q34.l.eu.2	114	-0.58	0.59	-0.3	1.14	4.31	1.85	5.08	-0.48	Y	N	N
CesA1	CELLULOSE SYNTHASE 1	F0F0A3T01A31VD.l.eu.2	169	-0.94	0.21	-0.41	-1.5	2.65	-0.43	n.a. (+)	-3.6	N	N	N
CCoAOMT1	caffeoyl-CoA 3-O-methyltransferase, putative	F0F0A3T01A4G7A.l.eu.2	55	0.41	-0.44	-0.07	0.19	0.14	0.07	0.27	0.09	Y	Y	Y
RBCL	large subunit of RUBISCO	F0F0A3T01A5ANA.l.eu.2	233	-0.1	0.06	0.89	-0.21	0.97	-0.06	1.17	-0.26	N	N	N
CER6	CUTICULAR 1; catalytic	F0F0A3T01EOHZ5.l.eu.2	16	-0.39	0.56	-0.63	-3.03	2.11	-0.32	4.23	-3.13	N	N	N

* Significant fold-change according to DEGseq analysis, at a q-value threshold of 0.05.

a: fold-change could not be calculated because no reads were found for the genotype considered in either the IR or the NI sequencing set.

b: fold-change could not be calculated because no expression was reported for the NI treatment, for the genotype considered.

c: fold-change could not be calculated because no expression was reported for the IR treatment, for the genotype considered.
